# When Survival Meets Fear: A Quantitative Study on Generalized Anxiety, Depression and Quality of Life in Cancer Survivors from Portugal

**DOI:** 10.3390/healthcare13233156

**Published:** 2025-12-03

**Authors:** Andreia Carrança, Ana Torres, Paula Carvalho

**Affiliations:** 1 Department of Psychology and Education, University of Beira Interior, 6200-209 Covilhã, Portugal; ana.carla.torres@ubi.pt (A.T.); paula.carvalho@ubi.pt (P.C.); 2 RISE-Health, Department of Medical Sciences, University of Beira Interior, Av. Infante D. Henrique, 6200-506 Covilhã, Portugal; 3 Research Center in Sports Sciences, Health Sciences and Human Development (CIDESD), University of Beira Interior, 6201-001 Covilhã, Portugal

**Keywords:** cancer survivor, Portugal, beira interior, generalized anxiety, depressive symptoms, fear of disease progression, quality of life

## Abstract

**Background/Objectives**: Due to ongoing medical and scientific progress, the number of cancer survivors is steadily increasing worldwide. However, this population remains particularly vulnerable to experiencing psychopathological symptoms and Fear of Disease Progression, which is often associated with lower Quality of Life scores. The present study aims to evaluate these variables among cancer survivors of the Portuguese Beira Interior Region, providing insight into this understudied population. **Methods**: A cross-sectional study was conducted with a convenience sample of 55 cancer survivors from the Beira Interior Region (69.1% female; M = 62.27 years), assessed through a sociodemographic and clinical questionnaire, alongside the PHQ-9, GAD-7, FoP-Q-SF, and QLQ-C30 instruments. **Results**: Overall, 60.9% of participants exhibited depressive symptoms, 43.6% reported generalized anxiety, and 54.5% showed normative levels of Fear of Disease Progression. Significant differences in Fear of Disease Progression were observed between sexes and educational levels. Positive correlations emerged between Fear of Disease Progression and both generalized anxiety (r_s_ = 0.66) and depressive symptoms (r_s_ = 0.43), while all three variables were negatively associated with Quality of Life. Although individual predictors did not reach statistical significance, the combined model, including Fear of Disease Progression, depressive symptoms, and anxiety, explained 21% of the variance in Quality of Life. **Conclusions**: These findings underscore the importance of assessing and monitoring Fear of Disease Progression among cancer survivors to support the development of early and tailored interventions, given Fear of Disease Progression’s predictive role in generalized anxiety, depressive symptoms, and reduced Quality of Life in this population.

## 1. Introduction

When addressing an oncological case, several aspects must be considered, such as the type of cancer, its stage and the type of treatment involved [1,2]. Although cancer remains the second leading cause of death in Portugal, the number of cancer survivors has increased over the years, largely due to scientific progress [3]. However, the term “cancer survivor” lacks a universally agreed-upon definition. Considering the findings of a study that analyzed survivors’ understanding and identification with the concept [4], and in alignment with the objectives of the present study, we adopted the definition provided by the National Cancer Institute [NCI] [5] and the National Coalition for Cancer Survivorship [NCCS] [4]. According to this definition, a cancer survivor is any individual who has been diagnosed with cancer from the moment of diagnosis until the end of life. From the point of diagnosis, throughout treatment and even after its conclusion, cancer survivors are exposed to a range of stressors, including psychological, physical, financial and social challenges, that can affect various aspects of life, such as QoL and survivorship outcomes [6,7]. In this regard, studies show that cancer survivors present higher vulnerability to develop depressive symptoms and anxiety compared to the general population, since these emotional symptoms are frequently comorbid with cancer [6,8].

Usually linked to anxiety in cancer survivors is the concept of Fear of Disease Progression (FoP), one of the most prevalent symptoms of distress in this population [9]. According to Dinkel and Herschbach [10], FoP is an appropriate response to a real threat: the possibility of cancer progression or recurrence and all its biopsychosocial consequences.

These feelings are experienced at multiple levels—emotional, cognitive, behavioral, and psychological—and, when maladaptive, can affect various domains, including QoL, emotional regulations, decision-making, treatment adherence, and overall well-being [11,12,13].

Research has identified several predictors of FoP, including anxiety [9,14], depression [9,15], sex [15,16] and educational level [9,17].

Though FoP is often discussed in connection with anxiety, it is conceptually distinct from generalized anxiety. Generalized anxiety reflects a persistent and broad pattern of worry across multiple life domains, whereas FoP represents a response to a real and concrete threat, the possibility of cancer recurrence and worsening [10,11,18]. This distinction is clinically relevant because most existing studies address anxiety in general terms without specifying whether they refer to generalized anxiety or to cancer-specific fears [8,19,20,21,22,23]. Moreover, generalized anxiety involves persistent and uncontrollable worry that may amplify illness-related cognitions, thereby heightening vulnerability to FoP [18]. Such gaps justify the need to examine generalized anxiety more precisely in relation to FoP in cancer survivors.

In line with this, most reviewed studies rely on the Hospital Anxiety and Depression Scale (HADS) [24], which assesses broad dimensions of anxiety and depression in hospital settings, and may therefore overlook clinically significant worry patterns [8,19,20,21,22]. In contrast, GAD-7 provides a focused assessment of generalized anxiety symptoms, as its structure is based on the DSM-IV-TR diagnostic criteria and evaluates all characteristic features of generalized anxiety [25].

Given the well-documented vulnerability of cancer survivors to developing anxiety and depressive symptoms, and considering that these conditions frequently co-occur with FoP, these psychological responses warrant particular attention. Although conceptually distinct, they may amplify one another, contributing to increased psychological distress.

Furthermore, these symptoms have consistently been associated with poorer QoL, which, according to the World Health Organization (WHO) [26], reflects the individual’s perception of their position in life, within the context of their culture and value system, goals, expectations, and concerns. According to Kuang et al. [12] and Liu et al. [27], low QoL is among the most reported challenges faced by cancer survivors.

This association may be explained by the presence of distressing emotions and illness-related cognitions, the adverse effects of treatment, as well as the experience of FoP. In addition, studies report the existence of a relationship between symptoms of depression, anxiety, FoP, and lower QoL levels [14,27]. Taken together, the interaction between these variables forms a complex framework that profoundly influences psychosocial adjustment throughout survivorship.

However, despite the substantial body of literature, important gaps still remain. As previously noted, most existing studies do not assess generalized anxiety, relying instead on global or nonspecific indicators of anxiety. Additionally, although FoP, anxiety and depression have each been investigated in cancer survivors, there is still a relative lack of studies that examine these three dimensions simultaneously and explore their combined relationship with QoL in this specific population.

In the Portuguese context, research in psycho-oncology addressing the variables under study remains limited, although some noteworthy findings have been reported. For example, Cruz [28] found that high levels of FoP were associated with symptoms of depression and anxiety. Similarly, Marinho [29] in a study evaluating FoP in cancer survivors, reported prevalence rates ranging from 24% to 70%, which proved to have a negative impact on some biopsychosocial domains and, consequently, on the sample’s QoL. In addition, Monteiro [30], in a systematic literature review, found that resilience played a protective role against psychological distress, being a good predictor of QoL in cancer survivors. Regarding the Beira Interior region, no published data is currently available that explores these variables in local cancer survivors.

This population may present unique regional characteristics, such as generally lower educational levels [31], less frequent contact with advanced technological tools, work-related traits associated with predominantly rural occupations [32], and an overall older age profile [33]. These distinctive features not only have the potential to influence the study variables but also offer an opportunity to broaden and enrich the current research landscape in this field.

Clinically, findings from studies of this nature may enhance the quality of care by enabling the identification of key risk factors for the development of depressive symptoms, generalized anxiety and FoP. Such findings also support the improvement of targeted screening protocols for these clinical conditions. Additionally, this line of research underscores the need to address the specific characteristics and unmet psychosocial needs of cancer survivors from the Beira Interior region.

Therefore, the present study aims to assess FoP among cancer survivors in the Beira Interior region of Portugal, exploring its relationship with sociodemographic variables, psychopathological symptoms, more specifically, depressive symptoms, generalized anxiety, and QoL.

To this end, the following hypotheses were proposed: FoP would be significantly present in the sample, revealing differences in its expression according to sex, educational level, cancer stage, knowledge of the cancer stage and type of treatment received. Additionally, it was hypothesized that survivors with generalized anxiety and depressive symptoms would report higher levels of FoP, and that these variables would be associated with lower QoL among cancer survivors from the Beira Interior region.

## 2. Materials and Methods

A convenience sample was collected from a hospital setting in the region, as well as from a community-based program dedicated to this population, promoted by the Department of Sports Sciences of the University of Beira Interior. The samples consisted of 55 Portuguese Cancer Survivors from the Beira Interior region, of whom 69.1% were female (*n* = 38) and 30.9% were male (*n* = 17), with a mean age of 62.27 years (SD = 11.91).

As shown in Table 1, 56.4% of participants reported being married, and 38.2% were classified as belonging to a lower-middle socioeconomic status. With regard to educational qualifications, 25.5% had completed primary education, and an equal proportion (*n* = 14) reported having completed secondary education. Concerning current employment status, 45.5% of the sample were retired. Additional sociodemographic characteristics are presented in Table 1.

The inclusion criteria for the present study were: (1) age over 18 years; (2) a cancer diagnosis, regardless of tumor type or anatomical location; (3) residence in the Beira Interior region.

### 2.1. Measures

To address the outlined objectives, an evaluation protocol was developed, comprising the following instruments: (1) the Sociodemographic and Clinical Questionnaire; (2) the PHQ-9 (Patient Health Questionnaire) [34]; (3) the GAD-7 (Generalized Anxiety Disorder) [35]; (4) the FoP-Q-SF (Fear of Progression Questionnaire-Short Form) [36]; and (5) the EORTC QLQ C-30 (Quality of Life Questionnaire Core-30) [37].

#### 2.1.1. Sociodemographic and Clinical Questionnaire

This questionnaire was designed to collect comprehensive sociodemographic information, such as sex, age, marital status, socioeconomic status, educational qualifications and current professional status. It also aimed to characterize the oncological condition through questions regarding the date of diagnosis, cancer type, presence of recurrences and metastases, disease stage and phase, and types of treatments received. Additionally, it assessed prior psychological or psychiatric follow-up by inquiring about previous diagnoses of psychological or psychiatric conditions, whether these diagnoses preceded or followed the cancer diagnosis, the presence of brain injuries, other health problems, and any history of substance abuse or dependency. This information was obtained through self-report, with participants being asked whether they had ever received a diagnosis from a mental health professional and to specify the type of condition.

#### 2.1.2. Patient Health Questionnaire (PHQ-9)

This instrument was used to assess the presence of depressive symptoms, consisting of 9 clinical items, along with an additional item that evaluates the extent to which the respondent’s difficulties impact various areas of functioning [38]. The response format was based on a four-point Likert scale, resulting in a total score ranging from 0 to 27 [34,38]. To determine the severity of depressive symptoms, the original version of the instrument defines the following cut-off points: 0–4 (minimal symptoms); 5–9 (mild symptoms); 10–14 (moderate symptoms); 15–19 (severe-moderate symptoms); 20–27 (severe symptoms) [38]. The Portuguese validation study of the PHQ-9 reported good internal consistency (α = 0.86) [38], a finding that was confirmed in the present study (α = 0.88).

#### 2.1.3. Generalized Anxiety Disorder Scale (GAD-7)

To assess the presence of generalized anxiety symptoms, the GAD-7 questionnaire was applied [35]. It consists of seven items, with responses provided on a four-point Likert-type scale ranging from “not at all” to “almost every day” [25]. The total score is calculated by summing the individual item scores [25]. Cut-off points classify the severity of anxiety symptoms as: none/normal (0–4), medium (5–9), moderate (10–14), or severe (15–21) [25]. The Portuguese validation study reported good internal consistency (α = 0.88) [25], which was also confirmed in the present study (α = 0.90).

#### 2.1.4. Fear of Progression Questionnaire-Short Form (FoP-Q-SF)

The short form of this instrument consists of 12 items rated on a five-point Likert-type response scale [39]. The total score is obtained by summing the scores of all items, with higher scores indicating greater FoP [39]. The Portuguese validation study reported good internal consistency (α = 0.86) [39], a result that was corroborated in the present study (α = 0.91).

#### 2.1.5. Quality of Life Questionnaire Core-30 (QLQ C-30)

The EORTC QLQ C-30 is a 30-item instrument designed to provide a multidimensional assessment of QoL [40]. It comprises five functional scales, three symptom scales, a global health scale, and several single items assessing symptoms commonly reported by cancer survivors [37,40]. Its scoring system is based on a four-point Likert scale ranging from 1 (not at all) to 4 (very much). The Portuguese validation study demonstrated good internal consistency across most scales [40], a finding that was also confirmed in the present study, both at the individual scale level and for the overall instrument (α = 0.83).

### 2.2. Procedure

Prior to initiating data collection, ethical approval was obtained from the Ethics Committee of the Unidade Local de Saúde da Cova da Beira (Approval No. 17/2024, date of approval: 25 May 2024). Additionally, the study was also conducted in accordance with the Declaration of Helsinki.

Following this, the data collection process began, during which paper-based assessment protocols were distributed to cancer survivors attending the oncology service and to all participants in the survivorship program. All responses were used solely for research purposes and were always analyzed anonymously.

Data collection took place between June and July of 2024. Recruitment occurred in a routine clinical setting and in a community-based survivorship program. Participants were eligible regardless of their current treatment phase; specifically, individuals could be recruited while awaiting treatment, undergoing active treatment, in remission, experiencing disease recurrence, or receiving palliative care. These categories were recorded to ensure an accurate characterization of the clinical context in which participants were assessed.

### 2.3. Statistical Analysis

Statistical analyses were performed using IBM SPSS Statistics 28. Descriptive statistics were conducted (descriptive statistics, means, standard deviation, minimums and maximums), as well as tests of the internal consistency of each instrument using Cronbach’s Alpha. Assumptions of normality, homogeneity, and multicollinearity were tested using the Kolmogorov–Smirnov Test, Levene’s Test and Collinearity Diagnoses, respectively.

To test the outlined objectives, the following statistical procedures were performed: One-sample Student’s *t*-test, used to compare the mean values obtained in the present study with those reported in the Portuguese validation studies of the respective instruments; Independent samples Student’s *t*-test, applied to examine differences in FoP between sex and participants awareness of their disease stage; Spearman’s Correlation Coefficient, used to assess associations between: FoP and generalized anxiety, FoP and depression, FoP and disease stage, QoL and FoP, QoL and generalized anxiety and quality of life and depression; Kruskall-Wallis test, used to evaluate differences in fear of progression across symptom severity levels and treatment modalities; Mann–Whitney U test, applied to compare differences in FoP based on educational level; and Linear Regression Analysis, conducted to determine the extent to which the selected independent variables predicted QoL. Effect sizes for correlation coefficients followed the guidelines proposed by Cohen [41], which define coefficients of 0.10, 0.30 and 0.50 as indicating small, medium and high effect sizes, respectively.

## 3. Results

Regarding clinical aspects, 42.6% of the sample (*n* = 23) had received the oncological diagnosis within the past year, while 40.7% (*n* = 22) had been diagnosed between two and five years before completing the protocol. The most prevalent cancer types were breast cancer (*n* = 26; 47.3%), colon and rectal cancer (*n* = 13; 23.6%) and lung cancer (*n* = 7; 12.7%). Notably, 40% of the participants did not know their disease stage at the time of diagnosis (*n* = 22). During the data collection period, 38.9% of the sample were undergoing active treatment (*n* = 21), and 53.7% were in remission (*n* = 29). Regarding the types of treatment received, 16.4% had undergone surgery alone (*n* = 9), 23.6% reported receiving both surgery and chemotherapy (*n* = 13) and 16.4% had received both surgery and hormone therapy (*n* = 2). Concerning psychological and psychiatric care, over half of the sample (*n* = 43; 78.2%) indicated they had never received, nor were currently receiving, such support. All of these data are summarized in Table 2.

### 3.1. Depressive Symptoms

With regard to the PHQ-9, the scores obtained ranged from 0 to 27, with a mean of 6.75 (SD = 5.84). In terms of symptom severity categories, the most prevalent was minimal depressive symptomatology (*n* = 27; 49.1%), followed by mild depressive symptomatology (*n* = 12; 21.8%) and moderate depressive symptomatology (*n* = 11; 20%). A one-sample *t*-test revealed a statistically significant difference between the levels of depressive symptoms reported by the present sample and those observed in the validation study of the instrument for the Portuguese context [38], with the current sample exhibiting higher symptom levels (M = 4.92; SD = 4.63; *t* (54) = 2.32, *p* = 0.03, two-tailed).

### 3.2. Generalized Anxiety Symptoms

With respect to the GAD-7, scores ranged from 0 to 18, with a mean of 4.65 (SD = 4.72). In terms of symptom severity categories, most of the sample manifested normal generalized anxiety levels (*n* = 31; 56.4%), while 27.3% presented mild symptoms (*n* = 15) and 12.7% with moderate symptoms (*n* = 7). By performing a *t*-test of a sample, it was possible to verify that the present sample reported lower levels of generalized anxiety compared to those found in the sample assessed by Sousa and colleagues [25] (M = 15.7; SD = 4.6; *t* (54) = −17.374, *p* = 0.00, two-tailed).

### 3.3. Fear of Disease Progression

Through the application of the FoP-Q-SF, scores ranged between 12 and 54, yielding a mean of 32.18 (SD = 11.12). To analyze the expression of FoP within the sample, the mean and standard deviation values reported by Silva and colleagues [36] were used to establish categorical thresholds. Thus, 54.5% of the sample exhibited normative levels of FoP (*n* = 30). Furthermore, statistically significant differences were observed between the expression of FoP in the present study population and that reported by Silva and colleagues [39], with the latter showing a higher mean response (M = 40.4; SD = 9.15; *t* (54) = −5.48, *p* = 0.00, two-tailed).

### 3.4. Quality of Life

Results from the EORTC QLQ C-30 revealed that the overall QoL in the current sample had a mean score of 58.79 (SD = 22.01). A comparison with the sample evaluated by Pais-Ribeiro and colleagues [40] (M = 62.00; SD = 22.60) revealed a statistically non-significant difference (*t* (54) = −1.08, *p* = 0.28, two-tailed), with the present sample reporting slightly lower QoL levels.

### 3.5. Sociodemographic and Clinical Variables

Statistically significant sex differences were observed in the expression of FoP, with female participants reporting higher levels (M = 35.45; SD = 10.37) than males (M = 24.88; SD = 9.31; *t* (53) = 3.60, *p* = 0.00, two-tailed, *d* = 10.06). The mean difference of 10.57 indicates a greater FoP expression among female participants.

Regarding educational levels, significant differences were also observed between individuals with lower educational levels (up to the 1st cycle of primary education) and those with medium or higher educational levels (equal to or higher than the 2nd cycle of lower secondary education), with the last group presenting higher FoP scores (Md^low educational levels^ = 24.50, *n* = 16; Md^medium/high educational levels^ = 37.00, *n* = 39, U = 466.00, z = 2.86, *p* = 0.00).

An analysis of the relationship between generalized anxiety, depressive symptoms, and FoP revealed a moderate positive correlation between depressive symptoms and FoP (r_s_ = 0.43, *n* = 55, *p* < 0.00), accounting for 18% of the variance. Additionally, a strong positive correlation was found between generalized anxiety symptoms and FoP (r_s_ = 0.66, *n* = 55, *p* < 0.00), explaining 44% of the variance.

Furthermore, comparisons between symptom intensity categories were also established in relation to the expression of FoP, revealing no statistically significant differences among the depression severity groups (H (4), 8.47, *p* = 0.08). In contrast, statistically significant differences were identified among the anxiety severity groups (H (3), 21.13, *p* = 0.00), particularly between the normal and mild categories (H = −14.40, *p* = 0.03) and normal and moderate categories (H = −25.77, *p* = 0.00). The analysis of the relationship between disease stage and FoP revealed no statistically significant association between the two variables (r_s_ = −0.31, *n* = 33, *p* < 0.08). Similarly, no statistically significant differences were found between survivors who were aware of their disease stage and those who were not (M^Knew the stage^ = 33.91; SD = 10.62; M^Did not know the stage^ = 29.59; SD = 11.59; *t* (53) = −1.43, *p* = 0.16, two-tailed, *d* = 11.01). The type of treatment received did not yield statistically significant differences in the expression of FoP (H (12) = 17.79, *p* = 0.12).

Several correlations were observed between QoL, FoP, depressive symptoms and generalized anxiety. A moderate negative correlation was found between FoP and both generalized anxiety symptoms and QoL (FoP–QoL: r_s_ = −0.35, *n* = 55, *p* < 0.01; Generalized Anxiety-QoL: r_s_ = −0.41, *n* = 55, *p* < 0.00). In contrast, a strong negative correlation was found between depression symptoms and QoL (Depressive Symptoms–QoL: r_s_ = −0.50, *n* = 55, *p* < 0.00) All correlation coefficients between the variables are summarized in Table 3. The coefficient of determination indicated that 25% of the variation in QoL was explained by depressive symptoms, 16% by generalized anxiety and 12% by FoP. Moreover, although none of the independent variables had a significant individual effect on predicting QoL (β_PHQ_ = −0.32, *p* = 0.06; β_FoP_ = −0.25, *p* = 0.14; β_GAD_ = −0.01, *p* = 0.96), as shown in Table 4, the combined set of predictors significantly explained the variance of the dependent variable (F (3) = 4.58, *p* = 0.01), accounting for 21% of the overall variability.

Collinearity diagnostics indicated that multicollinearity was not present in the regression model. Although none of the individual predictors reached statistical significance, this result is likely influenced by the limited sample size, which reduces statistical power for detecting independent effects. Nevertheless, the combined model significantly predicted QoL, accounting for 21% of its variance.

## 4. Discussion

The present study aimed to understand FoP, investigating its relationship with sociodemographic factors, generalized anxiety and depressive symptoms, as well as the impact of these variables on the QoL of cancer survivors from the Beira Interior region.

Given the fact that FoP is one of the most prominent unmet needs among cancer survivors, it is essential to assess and understand the emotional burden inherent to this phenomenon [9,42]. In this regard, the current sample exhibited lower levels of FoP compared to those reported by Cruz [28], Liga Portuguesa Contra o Cancro [43], Machado [44] and Silva and colleagues [39] in their analyses of Portuguese cancer survivors. The heterogeneity of results across studies regarding FoP expression may be partially attributed to the use of different assessment instruments, each with distinct cut-off points [42]. Additionally, lifestyle habits characteristic of populations from specific regions may exert either a protective or detrimental influence on the experience of these symptoms. In this regard, future research could focus on assessing these variables, which may contribute to identifying protective factors and behaviors as well as facilitate their adoption by broader populations

Moreover, the vulnerability of this population to experiencing psychological distress, as suggested in previous studies [6,8,19], was corroborated in the present sample, with 60.9% exhibiting some degree of depressive symptoms, and 43.6% reporting symptoms of generalized anxiety.

Despite these high rates of depressive symptoms and generalized anxiety, 78.2% of participants reported never having accessed psychological support. This mismatch reveals a substantial unmet need in the region, possibly linked to limited access to specialists and stigma associated with seeking mental health care.

Regarding sex differences in FoP expression, although the relationship remains somewhat unclear, several studies have reported such differences [15,16,42], a pattern also observed among cancer survivors from the Beira Interior region. As a possible explanation for this trend, Pang and Humphris [16] proposed that females tend to express a greater need for psychosocial support, as they present higher prevalence and severity of psychological and psychiatric disorders, and are more open to seeking help and expressing their emotional experiences. The findings from the present study suggest that the difference in FoP between women and men is not only statistically but also clinically significant, reflecting substantial variation in perceived threat and emotional burden. This magnitude of difference underscores the need for sex-sensitive screening and assessment practices.

About the relationship between FoP and educational levels, when such an association is observed, two main interpretations have been posited. A positive association, as presented in this study, may suggest that higher levels of education and increased access to additional information about diagnosis, treatment options, and prognosis may contribute to the development of catastrophic thoughts that, consequently, are reflected in higher levels of FoP [45]. On the other hand, when a negative association is found, knowledge is considered protective, as the lack of information may foster catastrophic interpretations [11,46]. However, some studies report no association between these variables [28,47]. Regarding the association between disease stage and FoP, some results point to a positive relationship between these variables [11,17]. Halbach and colleagues [17] suggest that more advanced stages may be perceived by survivors as indicators of disease progression and poorer prognosis, which may increase FoP. However, in alignment with the present findings, other authors such as Calderon and colleagues [46] and Oztas and colleagues [47] report no significant association between the variables. Similarly, findings regarding the association between the type of treatment received and FoP do not reflect a clear and consistent trend. In the present study, no significant associations were observed, however, some investigations report a positive association between radiotherapy and FoP [48,49], potentially due to the emotional and physical impact of undergoing radiotherapy, a methodology of treatment that entails several side effects, which can be perceived as a constant reminder of the illness or even as indicators of deterioration or recurrence [48,49,50,51].

Another noteworthy aspect is that 40% of participants were unaware of their disease stage, a clinically significant finding that underscores a substantial gap in clinical communication and may hinder both informed decision-making and active engagement in their own care. This high percentage may also be partly explained by the relatively low educational levels observed in this sample, which can limit patients’ understanding of the information provided by healthcare professionals and contribute to reduced clarity regarding their clinical status. Improved communication strategies and patient education interventions may help reduce uncertainty and FoP.

Diverging from the previously discussed associations, the relationship between FoP, depression symptoms and anxiety is well-established in the literature, with numerous studies reporting a positive correlation between these variables [14,45]. Interestingly, research has consistently shown a stronger correlation between depressive symptoms and FoP than between anxiety and FoP, a trend also observed in the present study [15,26]. Although this relationship is well-supported, its underlying mechanisms remain unclear. Some authors hypothesize that traumatic experiences often inherent to cancer may foster the development of depressive symptoms, anxiety, and FoP [18]. In addition, FoP encompasses a set of symptoms characteristic of various psychopathological conditions, and its association with affective states may increase individuals’ vulnerability to psychological distress [9,26].

Nevertheless, as previously noted, the relationship between FoP and generalized anxiety remains neglected. Most studies fail to specify which domains of the broad construct of anxiety are under investigation, potentially leading to ambiguity regarding the concept itself and the specific dimensions assessed. Moreover, the frequent use of assessment tools that evaluate only general or undifferentiated aspects of anxiety limits the ability to draw conclusions about this association. Unlike previous Portuguese studies that relied on broad or non-specific measures, such as HADS, the present study examined generalized anxiety specifically as operationalized by the GAD-7. This approach enables clearer differentiation between diffuse, chronic worry and situational cancer-related fear, thereby offering more refined insights for clinical screening and the design of targeted interventions. Given the substantial impact that generalized anxiety can wield on individuals’ lives, particularly when compounded by other sources of distress, a deeper and more precise understanding of this relationship with FoP is essential to mitigate its effects and develop suitable clinical interventions. Future research should prioritize the inclusion of disorder-specific measures to more accurately characterize anxiety profiles among cancer survivors and to inform tailored assessment and intervention strategies.

When interpreting these findings within the broader context of the study, it is important to note that although the overall regression model was significant, none of the individual predictors reached statistical significance. This pattern may indicate conceptual and statistical overlap among depressive symptoms, generalized anxiety, and FoP or it may be attributed to the limited sample size and consequent reduced statistical power. These constructs are known to co-occur as components of broader cancer-related distress, which may limit the ability to isolate unique predictive contributions.

Since QoL represents a subjective evaluation of one’s position in life, reflecting the expectations and concerns of the subject [24], it is likely to be compromised in this population due to the emotional and psychological burden related to the oncological condition. Indeed, several studies confirm this trend [6,12,25]. Following a more extensive approach, some investigations have explored the relationship between QoL, depressive and anxiety symptoms, and FoP, consistently revealing a negative association between variables [44,50]. The present findings are consistent with this trend. While some studies analyzed associations among these variables, few studies have assessed the individual predictive power of each. Apart from Mahendran and colleagues [45], whose findings align with those obtained in the present study, most research addressing these predictive relationships is more than a decade old. This underscores the importance of further exploring the interaction between these and other factors, clarifying the specific and individual contribution of each, in order to better identify individuals at higher risk and their respective needs.

The limitations identified in this study allow for the delineation of clear and meaningful directions for future research. The cross-sectional design prevents the establishment of any causal and/or temporal relationships among FoP, generalized anxiety, and depressive symptoms. Therefore, longitudinal studies are needed to clarify how these variables evolve over time and to identify periods of heightened vulnerability. Likewise, the limited representativeness of the sample, and particularly its marked heterogeneity regarding cancer type, time since diagnosis, and disease stage, constitutes a constraint for the interpretation of the findings, given that these clinical variables are known to be associated with FoP, and their variability within a small sample may have obscured differences that might otherwise emerge. Additionally, regarding the sample size, it was not possible to determine a representative sample size for this population due to the lack of publicly available data. Based on sample sizes used in comparable studies from other Portuguese regions, the present sample–although limited–meets commonly accepted minimum thresholds for exploratory observational studies (*n* ≥ 30). This underscores the need for larger, stratified samples that enable comparisons between subgroups. It is also essential to further investigate the specific role of generalized anxiety in FoP, as this dimension remains insufficiently explored in the current literature; future studies may benefit from mixed-method approaches or more robust predictive modelling to isolate its unique contribution. Finally, incorporating additional variables, such as coping strategies and resilience, which may support the development of more precise and culturally attuned interventions, ultimately guides clinical practice toward the actual needs of Portuguese cancer survivors.

## 5. Conclusions

This study found that cancer survivors from the Beira Interior region exhibit, for the most part, normative levels of FoP, with higher scores observed among female participants, those with medium to high educational levels, and individuals presenting symptoms of generalized anxiety and depression. In addition, it was possible to determine the nature and strength of the relationship between these variables and QoL within this population.

Based on the results obtained in the present study, it is possible to advocate for the following clinical recommendations: routine screening for depressive symptoms, generalized anxiety, and FoP should be implemented in cancer survivorship care, with particular attention to women and survivors with medium to high educational levels. Such screening practices would facilitate the early identification of individuals at higher risk for psychological distress, allowing healthcare professionals to provide timely and targeted interventions. Additionally, integrating systematic psychosocial assessment into survivorship follow-up may contribute to more comprehensive, patient-centered care, ultimately improving overall quality of life and supporting long-term emotional adjustment among cancer survivors.

Future research should pursue several complementary directions to expand the understanding of FoP and its related psychological processes among cancer survivors. Longitudinal studies are essential to map FoP trajectories over time and to clarify how fluctuations in depressive symptoms and generalized anxiety interact with survivorship experiences. Additionally, qualitative research focusing on region-specific protective factors may provide valuable insights into coping resources that are not captured through quantitative measures. The development and evaluation of tailored psychological intervention trials for this population would further contribute to identifying effective strategies for reducing FoP and enhancing QoL. Finally, research examining the role of physician-patient communication in shaping emotional adjustment and illness perceptions could offer important guidance for improving clinical interactions and optimizing psychosocial outcomes in survivorship care.

Given that 78.2% participants with significant symptoms had never accessed psychological support, strengthening psycho-oncological services in the region is an urgent public health priority.

## Figures and Tables

**Table 1:** Sociodemographic Data of the Sample.

	*n*	%
**Marital Status**		
Single	5	9.1
Married	31	56.4
Common-law union	6	10.9
Divorced or Separated	8	14.5
Widowed	5	9.1
**Socioeconomic Status**		
Low	9	16.4
Low-Medium	21	38.2
Medium	15	27.3
Medium-High	4	7.3
High	6	10.9
**Educational Attainments**		
Less than 4 years of education	2	3.6
Primary Education (1st cycle)	14	25.5
Lower Secondary Education (2nd cycle)	2	3.6
Upper Secondary Education (3rd cycle)	9	16.4
High School Education	14	25.5
Bachelor’s Degree Completed	9	16.4
Master’s Degree Completed	3	5.5
Doctorate Degree Completed	2	3.6
**Current employment status**		
Employed	16	27.3
On Medical Leave	12	21.8
Unemployed	2	3.6
Retired	25	45.5
Other	1	1.8

**Table 2:** Clinical Profile and Mental Health Care Characteristics of Participants.

	Category	*n*	%
Cancer Type	Breast	26	47.3
	Colon and Rectal	13	23.6
	Lung	7	12.7
	Others	9	16.4
Treatment Status	Active Treatment	21	38.9
	Remission	29	53.7
	Others	5	7.4
Psychological Care Data	Never received	43	78.2
	Has received/Currently receiving	12	21.8

**Table 3:** Spearman Correlation Matrix Between Study Variables.

	PHQ	GAD	FOP	QLQ
PHQ	-	-	0.43	−0.50
GAD	-	-	0.66	−0.41
FoP	0.43	0.66	-	−0.35
QoL	−0.50	−0.41	−0.35	-

**Table 4:** Results of the Linear Regression Analysis.

	QoL						
	β	Standar Error	*t*	*p*	*b*	*VIF*	Confidence Interval (95%)
PHQ	−0.32	0.63	−1.91	0.06	0.36	1.81	[−2.46; 0.06]
FoP	−0.25	0.33	−1.49	0.14	0.17	1.79	[−1.15; 0.17]
GAD	0.01	0.97	0.05	0.96	0.03	2.77	[−1.89; 1.99]

## Data Availability

The datasets presented in this study are available on request from the corresponding author due to privacy and ethical considerations, as they contain information that could potentially compromise the confidentiality and anonymity of the participants.

## Abbreviations

The following abbreviations are used in this manuscript:

EORTC	European Organization for Research and Treatment of Cancer
FoP	Fear of Disease Progression
FoP-Q-SF	Fear of Progression Questionnaire-Short Form
GAD-7	Generalized Anxiety Disorder
NCI	National Cancer Institute
NCCS	National Coalition for Cancer Survivorship
NHS	National Health Service
OECD	Organization for Economic Cooperation and Development
PHQ-9	Patient Health Questionnaire
QLQ C-30	Quality of Life Questionnaire Core-30
QoL	Quality of Life
SNS	Serviço Nacional de Saúde
WHO	World Health Organization
